# Community perspective on feasibility of malaria mass drug administration: a qualitative evidence from the Eastern Region of Ghana

**DOI:** 10.1186/s12936-026-05979-w

**Published:** 2026-06-10

**Authors:** Ignatius Cheng Ndong, Chuo Ennestine Chu, Ndong Henry Ndang, Collins Attuah Baah, George Asumah Adu, Emily Amponsah, Nana Yaw Peprah, Keziah Malm, Olumide Ogundahunsi, Collins Stephen Ahorlu, Alfred Amambua-Ngwa

**Affiliations:** 1 MRC Unit The Gambia at London School of Hygiene and Tropical Medicine, Banjul, Gambia; 2 Noguchi Memorial Institute for Medical Research, College of Health Sciences, University of Ghana, Accra, Ghana; 3 Department of Health Economics Policy and Management, Catholic University of Cameroon, Bamenda, Cameroon; 4 Njongndong Foundation, Yaoundé, Cameroon; 5 Department of Computer Engineering, University of Buea, Buea, Cameroon; 6 National Malaria Elimination Programme, Ministry of Health, Accra, Ghana; 7 Ghana Health Service, Ministry of Health, Accra, Ghana; 8 University of Medical Sciences, Ondo, Nigeria

**Keywords:** Malaria, Mass Drug Administration (MDA), Ghana, Community perceptions, Qualitative research, Public health

## Abstract

**Background:**

Malaria remains the leading cause of morbidity and mortality in Ghana, particularly in rural high-burden communities. Mass drug administration (MDA) has re-emerged as a complementary strategy to reduce transmission, yet community acceptability and implementation challenges remain underexplored.

**Methods:**

We conducted a cross-sectional study in the Pokrom subdistrict following pilot mass drug administration (MDA) interventions. Data were collected through eleven focus group discussions (FGDs), nine with community members and two with healthcare providers, comprising 97 participants, as well as eight in-depth interviews (IDIs) with assemblymen, healthcare providers and personnel of malaria elimination programme. Thes were complemented by field notes. Participants were purposively selected from individuals who had participated in the MDA interventions and provided informed consent. This study explored community perceptions of malaria burden, perceived MDA effectiveness, reported adverse events, health-seeking behaviour, financial implications and acceptability. Transcripts were analysed thematically using a hybrid inductive–deductive approach informed by reflexive thematic analysis.

**Results:**

Malaria was widely identified as the most pressing health concern in the subdistrict prior to the MDA intervention, with frequent hospitalisation and substantial household expenditure on treatment reported. Following the MDA interventions, notable reductions in malaria episodes were described, with some households transitioning from frequent infections to rare or no illness. These perceived improvements were accompanied by decreased healthcare-related costs, indicating important financial and economic benefits. Though adverse events, including dizziness, abdominal pain, and weakness, were reported, they were generally perceived as mild and transient. Initial challenges included inadequate community sensitization and negative messaging from some health workers, which temporarily affected uptake. Despite these concerns, willingness to participate in future MDA rounds remained high among community members, with recommendations emphasizing continued sensitization, strengthened drug delivery systems, and enhanced monitoring of side effects.

**Conclusions:**

The MDA interventions were widely perceived as beneficial in reducing malaria burden and associated household healthcare costs in rural Ghana. Consistent with the Theoretical Framework of Acceptability, perceived effectiveness and economic benefits outweighed the burden of transient adverse events, contributing to perceived high community acceptability. Strengthening communication, pharmacovigilance, and community engagement could be critical for sustaining uptake and supporting the potential scale-up of MDA in high-burden malaria settings.

**Supplementary Information:**

The online version contains supplementary material available at 10.1186/s12936-026-05979-w.

## Background

Malaria remains a leading cause of morbidity and mortality across sub-Saharan Africa and continues to impose a heavy health and economic burden in Ghana [1, 2]. Globally, an estimated 249 million malaria cases and 608,000 deaths were reported in 2022, with Africa accounting for approximately 94% of cases and 95% of deaths, underscoring the region’s disproportionate burden and the need for intensified, context-adapted interventions [3, 4].

In Ghana, malaria remains the principal cause of outpatient attendance and a major driver of paediatric admissions and household health expenditure, despite gains achieved through insecticide-treated nets (ITNs), indoor residual spraying (IRS), seasonal malaria chemoprevention (SMC), rapid diagnostic testing, artemisinin-based combination therapies (ACTs), and the recent rollout of the vaccines against malaria [5–7]. Rural communities continue to face high transmission intensity, frequent febrile illness, and substantial direct and indirect costs related to malaria treatment and lost productivity [8–10].

Mass drug administration (MDA), the administration of effective antimalarial medicines to entire populations or defined risk groups regardless of individual infection status has re-emerged as a potentially powerful, time-limited intervention to reduce parasite prevalence and interrupt transmission when combined with other control measures [11–14]. Evidence from clinical trials and programmatic experiences across Africa demonstrates that MDA can significantly reduce parasitaemia, clinical malaria episodes, and hospital visits in the short term, although its long-term effectiveness depends on achieving high coverage, repeated rounds, optimal drug regimens, and integration with vector control [15–19].

Community acceptance, pharmacovigilance, and effective communication are critical determinants of MDA success [20–24]. Qualitative studies from West Africa highlight the importance of trusted local volunteers, transparent sensitization campaigns, and responsive systems for managing adverse events in fostering high adherence [25–29]. Conversely, misinformation, fear of side effects, and lack of community engagement have been shown to undermine participation and programme effectiveness [30].

This report present findings from FGDs and IDIs complemented by field notes conducted in rural Ghanaian communities in the Pokrom sub district following five rounds of MDA implementation. The study explores community perceptions of malaria burden, experiences with MDA, perceived effectiveness of the medicine, reported side effects, treatment-seeking behaviour, financial implications and suggestions for programme improvement by community members and opinion leaders. The work aims to contribute towards informing policymaking and programme implementation on the feasibility, acceptability, and operational considerations required for effective MDA rollout in Ghana.

## Methods

### Study design and theoretical framework

This cross-sectional qualitative study was conducted to explore community perceptions and experiences following five rounds of a pilot MDA intervention implemented from December 2023 and August 2024 in the Pokrom subdistrict, Ghana. The study employed focus group discussions (FGDs) and in-depth interviews (IDIs), complemented by field notes, to capture community and health system perspectives on malaria burden, the perceived effectiveness of the MDA intervention, reported adverse events, health-seeking behaviour, financial implications and programme acceptability.

The study was conceptually informed by the Health Belief Model, which examines how individuals’ perceptions of disease risk, perceived benefits of interventions, perceived barriers, and cues to action influence health behaviour and intervention uptake [31]. This framework guided the development of the FGD and IDI guides and interpretation of findings related to community acceptance of the MDA intervention.

#### Research team characteristics

The research team consisted of experienced qualitative researchers and malaria specialists. Data collection was supervised by Dr. Ndong Ignatius and Prof. Collins Ahorlu, both of whom have extensive experience conducting malaria-related qualitative research in Ghana. The research team was made up of both males and females. FGDs and IDIs were facilitated by trained qualitative research assistants fluent in English and at least one of the dominant local languages (Twi or Ewe). Prior to the study, the research assistants underwent training on qualitative interviewing techniques, ethical research conduct, and the study objectives to ensure consistency in facilitation and data collection.

#### Relationship with participants

Participants were community members who had previously participated in the MDA intervention. The health personnel worked at the Pokrom health facility while the district health service and the NMEP provide the administrative oversight. While the research team had prior professional engagement with malaria research in the region, facilitators did not have established personal relationships with participants before the study. At the beginning of each discussion or interview, facilitators introduced themselves, explained the purpose of the study, and emphasized that participation was voluntary by taking participants through informed consent and signed agreement to participate in the study.

#### Study setting

The study was conducted in the Pokrom subdistrict in the Easter Region of Ghana [32] Pokrom is one of four sub-districts of the Akwapim South District. The district is served by four health centres and 22 Community-based Health Planning and Services (CHPS) compounds, with malaria being the leading cause of morbidity and mortality [33]. The Pokrom Health Centre, one of the oldest in the district, consistently reports malaria as the most common outpatient condition and serves as a focal site for the MDA intervention implementation, making the sub-district an appropriate setting for assessing community perspectives.

Participants were recruited from nine intervention communities: Pokrom, Amamfrom, Atakrom, Kwesi Dei, Nkumkrum, Dumpong, Yaw Dudu/Kwesi Krom, Adukrom/Obengkrom, and Kwamento. FGDs were conducted in community meeting venues such as church halls or school halls that were easily accessible and familiar to participants. Findings from the study could provide valuable insights into the community acceptability and effectiveness of MDA interventions and the overall health dynamics engendered within the participating communities.

#### Participant selection/sampling strategy

Participants were purposively sampled to capture diverse perspectives across communities, age groups, and gender [34, 35]. Community members who had participated in the repeated MDA intervention were eligible for inclusion. Five rounds of MDA using dihydroartemisinin-piperaquine delivered by community health volunteers (CHVs) through bi-monthly household visits. The nurses at the health facility who took part in the FDGs were working at the Pokrom health Centre when the MDA was implemented. Eleven FGDs (nine with community members and two with healthcare worker). A total of 97 participants were recruited for FGDs across nine communities, with approximately 8–12 participants per group (Table 1). Twelve participants were recruited per FGD; however, 11 individuals were unable to attend. This included adult men and women, caregivers of children, and health personnel from the Pokrom health facility, with ages ranging from 16 to 82 years.

**Table 1:** Characteristics of participants in FGD and IDI

	Number of participants								
Data Collection Technique	Female(n)			Male(n)			Total (n)	Mean age (n)	Age range (years)
	CR*	HW	Total	CR*	HW	Total			
FGD	49	7	56	32	9	41	97	44	16—82
IDI	2	2	4	2	2	4	8	4	40 -52

^*^CR Community Respondents, *HW* Health workers including officials of the National Malaria Elimination Programme

Additionally, eight key informants were purposively selected participated in IDIs, including four community assembly members, two personnel of the district health service, and two personnel from the National Malaria Elimination Programme. These key informants were aged between 40 and 52 years. Recruitment for FGDs and IDIs was conducted through face-to-face engagement within communities and workplaces. Participants were informed of the discussion venues one day in advance and provided with transport support to facilitate attendance. The occupation of the FDG participants ranged from farmers, petite traders, and nurses while the IDI participants included assemblymen, nurses, medical doctors, and health system administrators.

### Data collection

#### Interview guides

Semi-structured interview and discussion guides were developed based on previous studies examining community perspectives on malaria interventions in Ghana and West Africa [34, 36]. The guides explored themes including perceptions of malaria burden, experiences with the MDA intervention, perceived effectiveness of the medicines, reported adverse events, health-seeking behaviour, financial implications, and community acceptability. The guides were developed in English and translated into Twi and Ewe during data collectors’ training to ensure consistency in interpretation. Translation was contextual rather than literal, ensuring that questions conveyed equivalent meaning in the local languages. The tools were pre-tested in a community within the same district that was not included in the study. Feedback from the pilot testing informed revisions to improve clarity and consistency before data collection commenced.

#### Data collection procedures

Data collection took place between January and February 2025, six months after the MDA intervention. FGDs were conducted in community meeting venues such as church or school halls. IDIs with community assembly members were conducted in church halls, while interviews with district health personnel and staff from the National Malaria Elimination Programme were conducted via telephone. All FDGs were conducted in Twi or Ewe by experienced facilitators. All groups were mixed including males and female. All sessions were audio-recorded with participants’ consent and complemented with detailed field notes. Moderators encouraged participation from all group members and used probing techniques to explore emerging themes. Respondents were also encouraged to discuss and debate their views to provide deeper insights into shared and divergent perspectives. FGD sessions lasted between 45 and 60 min, while IDIs were conducted in English lasting between 30 and 45 min. Data collection continued until thematic saturation was reached, defined as the point at which no new themes or substantive insights emerged from additional interviews or discussions [37]. To enhance the accuracy of the data and ensure that the recorded information reflected participants’ views, audio recordings were replayed and reviewed with participants where necessary [38]. By the final FGDs and IDIs, responses were largely repetitive and reinforced previously identified patterns regarding perceptions of malaria burden, experiences with the MDA intervention, adverse events, financial implications, and community acceptability, indicating that sufficient depth and breadth of data had been achieved.

### Data management and analysis

Audio recordings were transcribed verbatim into English. Two trained qualitative researchers independently transcribed the recordings to ensure transcription accuracy. Transcripts were compared and discrepancies were resolved through discussion under the supervision of Collins Ahorlu. Regular debriefing sessions with research assistants were conducted during the data collection period to review emerging findings and ensure consistency in data collection. Data were analysed thematically using a hybrid inductive–deductive approach informed by the reflexive thematic analysis framework developed by Virginia Braun and Victoria Clarke [39].

An initial coding framework was developed based on the study objectives and interview guides, while additional codes were generated inductively as new themes emerged from the data. Coding was conducted independently by two researchers, and discrepancies were resolved through discussion until consensus was reached. The final codes were compiled into a codebook [37].

**Fig. 1:**
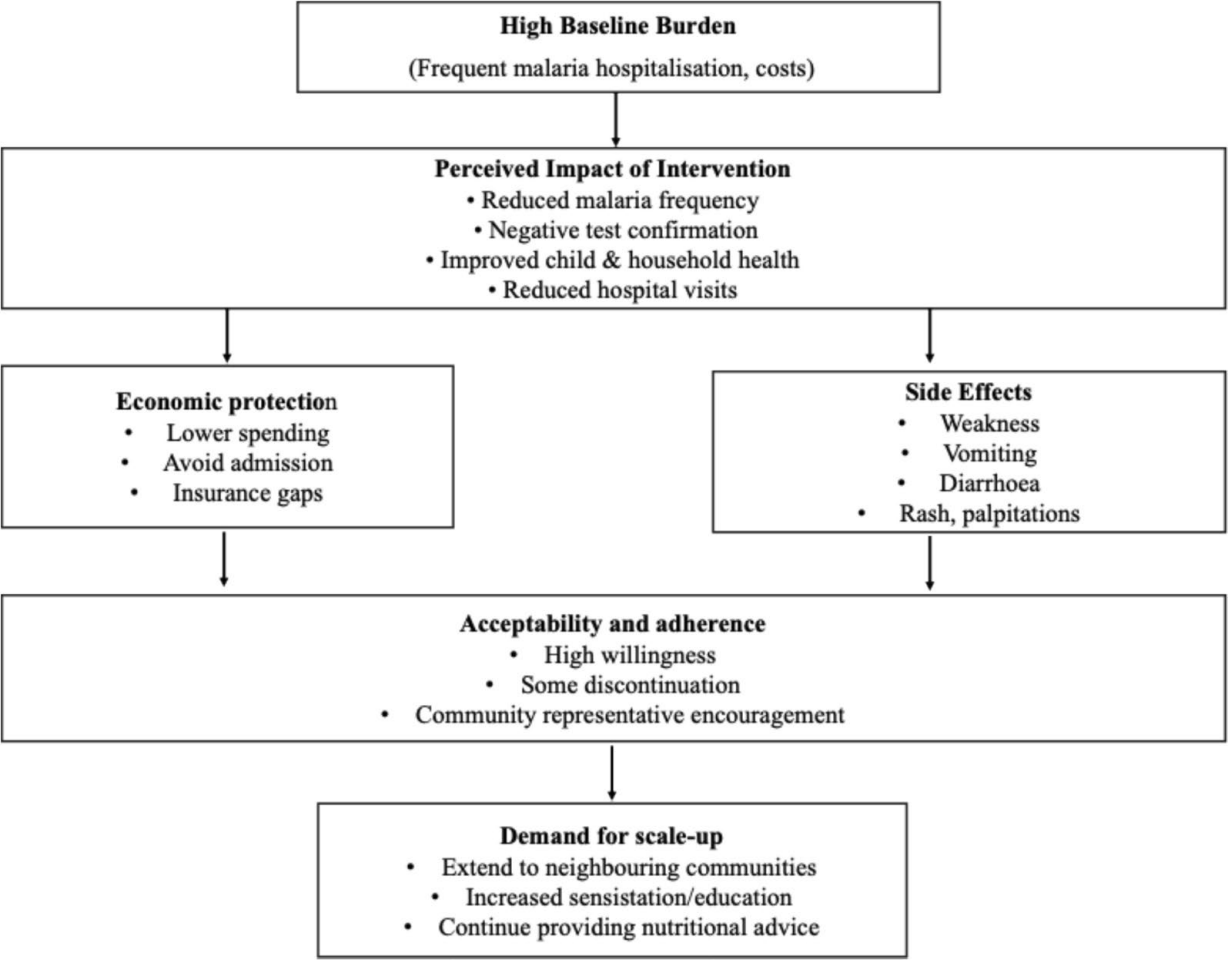
Conceptual Framework of the Health Belief Model illustrating the community perceived impact of malaria MDA interventions in Ghana**.** Perceived malaria reduction and economic protection outweighed transient side effects, leading to strong acceptability and demand for expansion

All transcripts were imported into NVivo version 12 for data management and coding. Themes were identified through thematic content analysis [40] and compared across communities to identify similarities and contextual differences in perceptions of the MDA intervention. To enhance analytical rigor, multiple data sources (FGDs, IDIs, and field notes) were triangulated [41], and coding decisions were discussed among members of the research team to ensure consistency and interpretive validity. Representative participant quotations were selected to illustrate key themes while maintaining participant confidentiality. This study explored community experiences and perceptions following a pilot MDA intervention in rural Ghana, guided by the Theoretical Framework of the Health Belief Model (Fig. 1). The participants were provided feedback following the transcription and analysis of the data.

## Results

A total of 11 FGDs (9 with community members and 2 with healthcare providers) and 8 in-depth interviews were conducted to explore perceptions and experiences following the pilot MDA intervention in the Pokrom subdistrict. Across all data sources, participants consistently described malaria as a substantial health and economic burden prior to the intervention, characterized by frequent illness episodes, repeated healthcare visits, and significant treatment-related costs, particularly among households with young children (Fig. 1). With an estimated population of 6000, five rounds of MDA were delivered in the intervention arm and courage increased from 68.4% in MDA round 1 to through 94.7% in MDA round 3 to 97.8MDA in round 5. This indicates a rapid scale-up and sustained coverage across successive rounds.

### Thematic map

Participants widely perceived the MDA intervention as effective, reporting noticeable reductions in malaria episodes, improved health status, and fewer hospital visits. These health gains were often associated with reduced financial expenditures on treatment and transport, highlighting important financial and economic benefits. Although mild and transient adverse events (including temporary weakness, dizziness, vomiting, or diarrhoea) were highlighted, these were generally considered acceptable, with most respondents indicating that the benefits of the intervention outweighed the side effects.

Overall, the findings seems to demonstrate strong community acceptance and support for the MDA programme, with many participants expressing willingness to continue participation and recommending its expansion. These qualitative insights underscore the perceived effectiveness, financial and economic relief, as well as social acceptability of the intervention, complementing the study’s quantitative outcomes (forthcoming).

### Perceived burden of malaria and other health problems

Across study communities, malaria was consistently described as the most significant health problem, often exacerbated during the rainy season. Participants also mentioned joint pains, hypertension and other chronic conditions as additional concerns.

*“Malaria is the leading health problem we have in this community*.” (P1, Adukrom).

*“Pain in the joins, waist, knee and legs, hypertension… are also health problems we face.” *(P6, Kwame Ntow).

### Awareness and views on pre-existing interventions

Concerning what interventions have been introduced in the community before the introduction of the MDA, while some community members acknowledged that they were aware of ITN distribution, others did not consider that as an intervention indicating that the introduction of the new medicine has significantly helped them. They maintained that the new programme led by community volunteers is particularly appreciated.

*“Apart from mosquito nets, the only intervention… is the medicine you have been bringing through the volunteers.”* (P2, Adukrom).

*“There has not been any intervention aside your drug distribution. They (MDA team) brought us medicine and due to its effectiveness […] we do not go to the hospital anymore.”* (P6, Nkumkrum).

### Perceived effectiveness of the MDA intervention

Participants overwhelmingly reported substantial reductions in malaria episodes, hospital visits, and household illness since MDA implementation. Many described dramatic changes, shifting from frequent illness to rarely getting sick. The community members seem to feel more empowered and believed that they are healthier now than before.

*“Since you started treating us, it has helped to reduce the malaria in the community.”* (P9, Dumpong).

*“I used to get malaria almost every 2 weeks, but we don’t see it again since we started using the medicine.”* (P7, Pokrom).

*“How I have suffered from malaria, you can inquire from the nurses. […], I and my children used to visit the hospital very often but now it is different. We do not fall sick very often anymore; the medicine has really been helpful to us. They should bring more.* (P5, Yaw Duodo).

Health workers corroborated this, describing visible declines in malaria-related attendance at the clinic as captured below:

*“This MDA programme is very effective… just looking at the attendance in the health facility, it has dropped significantly.”* (IDI, Nurse, Pokrom).

### Changes in health-seeking behaviour

Timeliness of care-seeking seems to vary from family to family. While some participants sought care early, others delayed or self-medicated. Despite this heterogeneity, MDA was perceived as reducing need for frequent health facility visits. These views were aptly represented in the following narratives:

*“If the illness persists for 3 days… I run to the hospital.”* (P4, Atakrom).

*“You will be taking some medication at home until the illness strikes you down. Then you will go to the hospital”* (P1, Dumpong).

### Improvement in overall health and well-being

MDA was widely associated with improved wellbeing, fewer symptoms (fever, chills, headache), and enhanced quality of life. Parents perceived improved child health and fewer school absences as impact of the MDA intervention. These views are represented in the following narratives:

*“My grandchildren do not fall sick again because of the MDA.” *(P6, Kwame Ntow).

*“My grand children do not longer stay away from school due to malaria.” *(P8, Pokrom).

### Community MDA acceptability factors

Several components of the intervention were perceived to have been consistently praised by participants the most including: 1) the free medication, 2) the effectiveness of the drugs used 3) the commitment and kindness of the community volunteers, 4) follow up visits post-treatment, 5) messages were clearly communicated and respectful engagement, and 6) drop in school absenteeism due to malaria. These perceptions collectively fostered trust and high acceptability of the MDA intervention as seen below:

*“The community volunteers’ commitment has impressed me so much.”* (P1, Adukrom).

*“They take their time to explain to you why the medicine is important.”* (P1, Pokrom).

### Perceived side effects and implementation challenges

Although most participants tolerated the medicine well, some experienced mild side effects including vomiting, stomach upset, hunger, dizziness, and increased urination. Adherence to the 3-h fasting rule before or after taking the medication was particularly challenging.

*“Some people vomit; some have stomach offset… you should intensify the education.”* (P6, Pokrom).

*“It is difficult to stay for the 3 h without eating after taking he medicine. It makes me feels very hungry shortly after taking it.”* (P6, Pokrom).

*“It caused my head to ache, but I later became okay.”* (P5, Yaw Duodo).

The MDA implementation initially faced misinformation and resistance, including negative messaging from some health workers. Working through the assemblymen, the project team was able to engage the community again and this enabled them to take ownership of the programme. These experiences highlight the importance of community engagement health worker alignment, and stronger communication strategies during MDA implementation. The following supports this view.

*“Some people from the health facility told us not to take the drug, that it was not good […], but we later realised it was not true through our assemblyman.”* (P1, Kwesi Dei),

*“It is my responsibility to provide good healthcare to my people by all means possible. So, we cannot let good programmes like the MDA pass by […] this explains why as the Assemblyman, I am leading the campaign. Our health is paramount, and you are concerned about it, therefore we must be very concerned too.”* (IDI, Assemblyman).

### Financial relief and economic benefits

Participants reported substantial reductions in healthcare spending, contributing to improved family finances and reduced economic stress.

*“We are healthy, now we do not go to the hospital to spend money.”* (P1, Adukro).

*“My finances are stable… I do not spend any money treating malaria again.”* (P2, Yaw Duodo).

The perceived reduction in malaria incidence seems to have shifted health-seeking norms, with fewer participants needing routine clinic visits. It also emerged that the reduced outpatient attendance observed at the health facilities posed challenges to health facilities, in terms of internal revenue generation. This position was highlighted by a healthcare provider:

*“The impact of this intervention is so high as malaria cases have greatly reduced. However, some of the health facilities may have to close if there is no alternative funding. Other diseases need to be funded.”* (IDI, District Health Office).

### Recommendations for improving the MDA

Participants suggested more education to engage communities, recruitment of additional volunteer, reduction of the interval between MDA rounds as well as the addition of other health interventions to address joint pain and other common health ailments.

*“There should be more education and engagement.”* (P4, Kwesi Dei).

*“You should add body and joint pain medication to the malaria drugs.”* (P8, Kwame Ntow).

*“You should get more volunteers to speed up the testing and treatment.”* (P7, Kwame Ntow).

### Other perceived benefits of the malaria MDA programme

Other educational messages from the programme were perceived to have contributed to improving the wellbeing of the community member. Participants revealed that sensitisation messages on the use of local iron-rich foods such as cassava leaves seemed to have improve their health especially children under 15 years of age as indicated by these quotes:

*“Since we started giving cassava leaves […] my child is no longer short of blood.”* (P5, Nkumkrum).

*“One thing that stands out for me about this programme is sensitisation message, when you educated us that we should consume cassava leaves to improve our children’s health. This was very important to my family* (P2, Pokrom).

Some women have been on family planning and stopped but could not regain their menstrual flow, report the resumption of the menstruation after taking the DHAP treatment.

*“After the period of my family planning injection expired, I expected that my menses should resume but it did not. So, I was very worried. But when I took this medicine, it started flowing again.* (P8, Pokrom)*”*

### Policy-level and health-system perspectives

The desire that the MDA programme be extended to other communities was relatively very high among participants. There was enthusiastic support for expanding the intervention to neighbouring communities, especially those lacking health facilities and to avoid the malaria spreading from those communities to their own communities where it is very low now.

*“It should be extended so others can benefit as well.”* (P6, Obengkrom).

*“Most villages around here don’t have health centres […] so this programme should be extended.”* (P10, Kwesi Dei).

Health managers emphasised the need for sustainable funding, especially as declining malaria cases impact facility internally generated finances. The National Malaria Elimination Programme (NMEP) expressed commitment to scaling MDA but noted challenges due to WHO guidance.

*“We believe in MDA […], we hope to scale it up.”* (IDI, NMEP Personnel).

*“WHO does not recommend MDA in high-endemic countries, so it is difficult to get funders.”* (IDI, NMEP Personnel).

These insights highlight the dichotomy between local evidence of effectiveness of malaria interventions and global policy constraints.

Consistently, health professionals in the different health facilities in the intervention communities, though appreciated the impact of the intervention, they express the need for alternative funding for infectious diseases other than malaria.

*“The impact of this intervention is so high as malaria cases have greatly reduced. However, some of the health facilities may have to close if there is no alternative funding. Other diseases need to be funded.”* (IDI, District Health Office).

*“We must work hard to improve adherence to the treatment protocols. We also need to strengthen the system because beyond malaria other diseases may come up”* (IDI, NMEP Personnel).

Despite the challenges of MDA interventions, the Ghana NMEP has taken steps to include MDA in its strategic plan as demonstrated in the quote below.

*Thank you very much for the opportunity […] and for piloting this intervention […]. The view of the NMEP is clearly stated in the strategic plan. In the current strategic plan, we hope to implement MDA in 21 districts earmarked for elimination. What this means is that we need the tools that can lead us to that point […] You will realise that you have gone very low (prevalence), but you have not been able to hit zero because there are surrounding districts and also because of the problem of hypnozoites. That is why in our current plan in addition to doing the MDA we are introducing primaquine with case management to help us clear hypnozoites and issues of gametocyte that have not been cleared even though the patient is well, […] you know. I am sure, we can hit ourselves almost at zero (prevalence).* (IDI, NMEP Personnel).

## Discussion

### Malaria as a persistent public health burden

The findings of the current study consistently described malaria as the predominant health problem in the communities, corroborating recent evidence from the World Health Organization that malaria remains a major contributor to morbidity in endemic regions [42]. These perceptions underscore the population’s longstanding vulnerability to malaria and the need for sustained interventions. In Ghana, malaria remains the most common outpatient diagnosis, accounting for approximately 38% of all outpatient visits and 27% of hospital admissions among children under 15 years [5]. Similarly, in West Africa, malaria continues to be a major driver of morbidity and mortality, with the region carrying a disproportionate share of the global malaria burden [3].

Although other conditions such as joint pain, hypertension, and skin problems were mentioned, malaria overshadowed them due to its recurrent nature, financial implications, and impact on productivity. This prioritisation is consistent with findings from sub-Sahara Africa, where communities similarly placed malaria above other health concerns [43–46].

### Effectiveness of the MDA intervention

Substantial reductions in malaria cases and hospital visits following the introduction of MDA were reported while some households migrated from experiencing monthly malaria to rare or no cases over the study period, illustrating perceived effectiveness. This corroborates findings from trials across Africa where community-wide antimalarial administration has been reported to significantly reduced parasite prevalence and febrile episodes [11, 47–50]. Despite the perceived reduction in malaria over the MDA implementation period, such reduction have been observed to be mostly transient, except the intervention is repeated over an extended period complemented with other control strategies [51, 52].

Globally, MDA has been shown to reduce malaria transmission in high-burden settings, especially when combined with other interventions such as ITNs and IRS [53–55]. In The Gambia, MDA with dihydroartemisinin–piperaquine reduced asymptomatic parasitaemia by over 60% [56].

Community testimonies from this study provide qualitative evidence supporting the quantitative outcomes of the intervention (forthcoming). Importantly, participants highlighted broader benefits beyond malaria, including improved household wellbeing, reduced out-of-pocket health expending, and enhanced financial savings. These findings are consistent with economic studies across sub-Sahara Africa showing that reducing malaria cases lowers catastrophic health expenditures and improves productivity [2, 57–61].

### Community engagement and volunteer role

Community representatives and volunteers were recognized as central to intervention success, owing to their efforts in drug distribution, follow-up, and addressing fears through information, education and communication strategies. Similar lessons have emerged from MDA programmes in different parts of Africa where community ownership and trust in local volunteers were critical for uptake [20, 62–64]. Participants also valued the free provision of medicines, which removed financial barriers and promoted equity, a finding supported by earlier studies showing that cost-free access enhances compliance in malaria interventions [65]. The fact that participants showed a sense of limited prior support in the community highlights their appreciation for the current MDA programme.

### Challenges: side effects and negative campaigns

Although many participants reported no adverse effects, some described transient vomiting, diarrhoea, dizziness, rash, or weakness. Side effects are a common challenge to MDA adherence globally, often worsened by inadequate sensitisation [3, 66]. These reported side effects did not deter participation due to strong community engagement [8]. A notable barrier perceived by participants in this study was negative messaging from some health workers, which initially undermined acceptance. Similar issues have been documented in different parts of Africa, where misinformation reduced uptake until local leaders intervened [16, 25, 28]. The pivotal role of assemblymen in countering misinformation highlights the importance of political and community leaderships’ involvement for sustaining MDA efforts [67].

### Health-seeking behaviour

The findings of this current study revealed variations in timeliness of care-seeking, with some individuals delaying hospital visits until symptoms worsened. This is consistent with patterns observed in sub-Sahara Africa and Southeast Asia, where self-medication and delayed treatment are common due to financial constraints, distance to facilities, or perceived low severity [2, 68, 69]. The perception of a reduction in the frequency of hospital visits following MDA interventions among participants was seen as both health and financial reliefs corroborating the findings from a study in sub-Sahara Africa [2, 22].

### Broader impacts on household finances and wellbeing

The fact that MDA intervention resulted in perceived financial relief is particularly important as participants reported that reduced hospital visits and treatments the saved money (up to 600 GHS per episode in some cases). This corroborates earlier findings suggesting that households in Ghana spend an average of USD 5–15 per malaria episode, which can escalate to catastrophic costs in severe cases [59]. By reducing illness episodes, the MDA interventions seem to have indirectly supported income stability, productivity, and quality of life, consistent with other findings from sub-Sahara Africa [22, 70, 71]. This is consistent with the fact that many individuals reported being “healthier than before” and credited the MDA for restoring household stability.

### Community recommendations for improvement

**Fig. 2:**
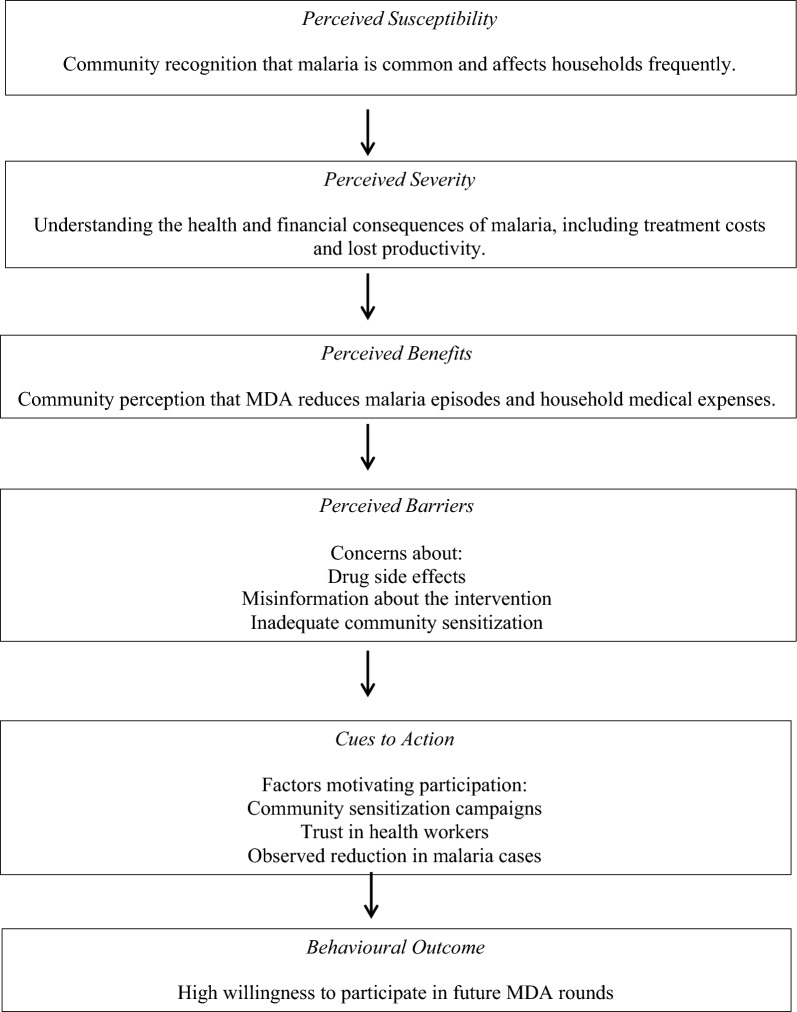
Conceptual Pathway: Qualitative findings were structured using the Health Belief Model, which explains how community perceptions influence participation in health interventions

The recommendations resultings from this study highlight the critical role of community perspectives in shaping the success and sustainability of malaria elimination strategies. Calls for enhanced community education reflect persistent concerns about misinformation and fears related to drug side effects, which have been widely reported in MDA programmes across sub-Saharan Africa and south East Asia [9, 14, 72–75]. These findings are consistent with the Health Belief Model (Fig. 2), which emphasizes that perceived barriers can limit uptake unless addressed through clear communication and trusted community engagement [31, 76, 77].

### Conceptual framework for MDA acceptability

The suggestion for more frequent drug distribution underscores the perceived benefits of sustained protection against malaria and aligns with experiences from MDA and seasonal malaria chemoprevention programmes [78–80]. However, such approaches must be balanced against operational feasibility and the risk of antimalarial resistance, as highlighted by the World Health Organization [78, 81, 82]. Participants also emphasized the need to expand MDA to underserved communities, reflecting persistent inequities in healthcare access that continue to drive malaria burden in rural settings [83–85].

Importantly, the recommendation to integrate MDA with services for other health conditions, such as hypertension and joint pain, reflects growing expectations for holistic, people-centred care. Evidence suggests that integrating vertical malaria interventions into broader primary healthcare systems can enhance acceptability, efficiency, and sustainability in resource-limited settings [86–89].

Finally, concerns raised by officials from the National Malaria Elimination Programme regarding alternative financing highlight a key challenge for sustaining malaria gains. As malaria burden declines, maintaining long-term political and financial commitment becomes critical [90–92]. Overall, these findings demonstrate that incorporating community-driven insights into programme design can improve uptake, enhance equity, and strengthen the long-term effectiveness of malaria elimination strategies.These align with WHO’s global strategy, which emphasizes integrating MDA with other community health services including strengthening communication strategies to ensure sustainability [3].

### Study limitations

This study has several limitations. First, the qualitative design relied on self-reported experiences and perceptions, which may be subject to recall bias or social desirability bias. Second, the study was conducted within a single subdistrict, which may limit the generalizability of the findings to other settings with different malaria transmission patterns or health system contexts. Third, although efforts were made to ensure diverse representation across communities, some invited participants were unable to attend the discussions, which may have limited the breadth of perspectives captured. Finally, interviews conducted by telephone with district health personnel may have limited opportunities to observe non-verbal cues compared with face-to-face interviews. Despite these limitations, the use of multiple qualitative methods, including FGDs, IDIs, complemented by field notes, strengthened the credibility and depth of the findings through triangulation of data sources.

## Conclusion

The findings of this qualitative study suggests that community-wide malaria mass drug administration was perceived to substantially reduce illness episodes, health care facility visits, and out-of-pocket expenditures, while improving household wellbeing and productivity. This report underscore the importance of strong community engagement, trusted volunteers, and responsive leadership in sustaining uptake, as well as the need to address side effects and misinformation promptly. Overall, MDA was viewed as both a health and economic intervention, reinforcing its potential role in high-burden settings such as rural Ghana.

## Supplementary Information

Additional file 1.

## Data Availability

The dataset used and analysed during the study is available from the corresponding author on reasonable request.
