# Supplementary material for: Community perspective on feasibility of malaria mass drug administration: a qualitative evidence from the Eastern Region of Ghana
Source: Malar J. 2026 Jun 10;25:235. doi: 10.1186/s12936-026-05979-w (PMC13251284; doi:10.1186/s12936-026-05979-w)
Supplement: Supplementary file 1 — Additional file 1. [file 12936_2026_5979_MOESM1_ESM.docx]

COREQ 32-Item Checklist Mapped to MDA Qualitaive work

The table below maps the checklist items to the sections of your manuscript.

| **COREQ Domain** | **Item Description** | **Where Addressed in Manuscript** |
| --- | --- | --- |
| ***Domain 1*** | ***Research Team and Reflexivity*** | |
| 1 | Interviewer/facilitator | Methods – Research team section |
| 2 | Credentials of researchers | Methods – Research team |
| 3 | Occupation | Methods – Research team |
| 4 | Gender of researchers | Methods – Research team description |
| 5 | Experience and training | Methods – Training of facilitators |
| 6 | Relationship with participants | Methods – Relationship with participants |
| 7 | Participant knowledge of interviewer | Methods – Data collection introduction |
| 8 | Interviewer characteristics | Methods – Reflexivity section |
| ***Domain 2*** | ***Study Design*** | |
| 9 | Methodological orientation | Methods – Reflexive thematic analysis |
| 10 | Sampling strategy | Methods – Participant selection |
| 11 | Method of approach | Methods – Recruitment |
| 12 | Sample size | Methods – Participants |
| 13 | Non-participation | Methods – Participant recruitment description |
| 14 | Setting of data collection | Methods – Study setting |
| 15 | Presence of non-participants | Methods – Data collection procedures |
| 16 | Description of sample | Table 1 – Participant characteristics |
| 17 | Interview guide | Methods – Data collection tools |
| 18 | Repeat interviews | Not applicable |
| 19 | Audio/visual recording | Methods – Data collection |
| 20 | Field notes | Methods – Data collection |
| 21 | Duration of interviews | Methods – Data collection |
| 22 | Data saturation | Methods – Data collection |
| 23 | Transcripts returned to participants | Methods – Data validation |
| ***Domain 3*** | ***Analysis and Findings*** | |
| 24 | Number of data coders | Methods – Data analysis |
| 25 | Coding framework | Methods – Codebook development |
| 26 | Derivation of themes | Methods – Hybrid inductive–deductive analysis |
| 27 | Software | Methods – NVivo |
| 28 | Participant checking | Methods – Validation process |
| 29 | Quotations presented | Results section |
| 30 | Consistency of data and findings | Results section |
| 31 | Clarity of major themes | Results section |
| 32 | Clarity of minor themes | Results section |
